# The impact of consuming different types of high-caloric fat diet on the metabolic status, liver, and aortic integrity in rats

**DOI:** 10.1038/s41598-024-68299-6

**Published:** 2024-08-10

**Authors:** Nardien Ekram Haliem Saleh, Mariam Yahia Ibrahim, Adel Hussein Saad, Elshymaa A. Abdel-Hakeem, Rabeh Khairy Saleh, Wagdy N. Habeeb

**Affiliations:** 1 Department of Medical Physiology, Faculty of Medicine, Minia University, El-Minia, 61511 Egypt; 2 Department of Pathology, Faculty of Medicine, Minia University, El-Minia, 61511 Egypt

**Keywords:** High caloric fat diet, Insulin resistance, Leptin, PUFAs, MUFAs, MCFAs, SCFAs, Biochemistry, Cell biology, Physiology, Biomarkers, Health care

## Abstract

Consumption of high-caloric diets contributes to the alarming number of overweight and obese individuals worldwide, which in turn leads to several diseases and multiple organ dysfunction. Not only has the number of calories taken per day but also the type of fat in the diet has an important impact on health. Accordingly, the purpose of the current study was to examine the impact of different types of high-caloric fat diets on the metabolic status and the integrity of the liver and aorta in albino rats. Adult male albino rats were divided into 6 groups: Control group, long chain-saturated fat group (SFD), long chain-monounsaturated fat (MUFAs) group, long chain-polyunsaturated fat (PUFAs) group, medium-chain fat (MCFAs) group, and short-chain fat (SCFAs) group. Body mass index (BMI), Lee index, and visceral fat amount were reported. Serum levels of insulin, liver transaminases, lipid profile, and different oxidative stress and inflammatory markers were evaluated. Homeostasis Model Assessment of Insulin Resistance (HOMA-IR), and adiponectin/leptin ratio were also calculated. Histopathological examinations of liver and aorta with Masson’s trichrome stain, and immune-staining for Nuclear Factor Erythroid-2-Related Factor-2 (Nrf2) were also done. SFD group showed significantly elevated liver transaminases, inflammatory markers, HOMA-IR, dyslipidemia, reduced adiponectin, and deficient anti-oxidative response compared to other groups together with disturbed hepatic and aortic architecture. Other treated groups showed an improvement. PUFAs group showed the highest level of improvement. Not all high-fat diets are hazardous. Diets rich in PUFAs, MUFAs, MCFAs, or SCFAs may protect against the hazards of high caloric diet.

## Introduction

An ideal daily requirement of calories normally varies depending on age, level of physical activity, and other factors. On average, the recommended daily caloric intake ranges from 1600 to 2200 per day for adult females and from 2000 to 3200 per day for adult males^1^*.* The 2015–2020 Dietary Guidelines for Americans offer some recommendations to avoid trans-fat, limit saturated fats to < 10% of calories per day, and replace them with healthier unsaturated fats^2^*.*

In high-caloric diet, the calories may exceed 4000 to 5000 cal a day. It may contain a higher fat or carbohydrate percentage than the normal range^3^. While fats have an important impact on health, the type of fat in the food should be considered as well. A high-fat diet may be rich in saturated or unsaturated fats^4^*.* In addition to the degree of saturation, there is an evidence to propose that the chain length of fatty acids is a major determinant of their effect^5^*.*

According to the chain length of fatty acids, they are classified into short-chain fatty acids (SCFAs), medium-chain fatty acids (MCFAs), and long-chain fatty acids (LCFAs). SCFAs are usually found in low proportions in the diet and are mainly produced by gut microbiota fermentation of dietary fibers^4^*.* MCFAs are found at high concentrations in food such as milk products, palm kernel oil, and coconut oil and their digestion is rapid and simple^6^. Full-fat dairy products such as butter are high in saturated LCFAs, whereas nuts, seeds, and vegetable oils are good sources of unsaturated LCFAs. Olive oil is very rich in monounsaturated fatty acids (MUFAs) especially oleic acid. While, eicosapentaenoic (EPA) and docosahexaenoic (DHA) are examples of omega-3 fatty acids; a specific type of polyunsaturated fatty acids (PUFAs) that are found in seafood^7^*.*

Saturated fatty acids originating from the triglyceride stores in adipose tissue are closely related to oxidative stress and a chronic inflammatory state that contributes to endothelium dysfunction and atherosclerosis of arteries including the aorta^8^. Inflammation induced by adipose tissue hypertrophy raises insulin resistance both locally and systemically, which enhances the co-morbidities associated with obesity. While, it was revealed that unsaturated fatty acids have a role in lowering inflammation, which in turn alleviates insulin resistance and reduces the risk of diabetes mellitus type II and cardiovascular disease in obese people^9^.

It is known that excess accumulation of fat especially in the visceral compartment (visceral obesity) is more strongly related to metabolic risk factors. Adipose tissue produces a variety of molecules known as “adipokines”, such as leptin, adiponectin, and resistin. In the case of obesity, adipose tissue hypertrophy and hyperplasia lead to alteration in adipokines which may play a role in the development of the metabolic syndrome. This is attributed to increased macrophage infiltration and production of pro-inflammatory molecules^10–12^.

One of the approaches of body defense against oxidative stress resulting from excessive accumulation of fat is targeting the nuclear erythroid 2-related factor 2 (Nrf2). Nrf2 binds to antioxidant response elements (AREs), which are found in the promoters of genes that express the antioxidant enzymes resulting in elimination of reactive oxygen species (ROS)^13^.

Previously, it was thought that excessive intake of fats, due to their high caloric value, is associated with an increased risk of obesity, metabolic syndrome, type II diabetes, and coronary heart disease, and could trigger hepatic steatosis, and fibrosis^14^. However, recently, the focus of research has shifted to the type of consumed fats, rather than their amount, that have different impacts on health according to their chain length of fatty acids and whether saturated or unsaturated^15^. Accordingly, the current study aims to compare the effect of high-caloric fat diets with different fatty acids’ chain lengths on metabolic status, and the liver and aortic integrity, and to explore whether all types of these fats have hazardous effects on health or not.

## Results

### Changes in final body mass index (BMI), Lee index, and visceral fat in different groups

Table 1 shows that final BMI, Lee index, and visceral fat amount were significantly increased in the SFD group as compared to other groups. The differences between levels of final BMI, Lee index and visceral fat in (MUFAs, PUFAs, MCFAs, and SCFAs) groups were insignificant.

**Table 1:** Initial and final BMI, final Lee index, and amount of visceral fat collected after dissection in different groups.

Groups (n = 7)						
Parameters	Control	SFD	MUFAs	PUFAs	MCFAs	SCFAs
Initial BMI (g/cm^2^)	0.52 ± 0.005	0.51 ± 0.01	0.52 ± 0.01	0.51 ± 0.01	0.51 ± 0.01	0.52 ± 0.01
Final BMI (g/cm^2^)	0.61 ± 0.01	0.74^a^ ± 0.02	0.62^b^ ± 0.01	0.64^b^ ± 0.01	0.63^b^ ± 0.01	0.63^b^ ± 0.01
Final Lee index (g/cm)	0.29 ± 0.01	0.34^a^ ± 0.03	0.3^b^ ± 0.02	0.31^b^ ± 0.02	0.3^b^ ± 0.01	0.31^b^ ± 0.02
Visceral fat (g)	2 ± 0.08	4.43^a^ ± 0.3	2.29^b^ ± 0.18	2.71^b^ ± 0.29	3^ab^ ± 0.36	2.79^b^ ± 0.34

Data represent mean ± S.E. of observations from seven animals. a: significant from the control group. b: significant from long saturated fat (SFD) group, c: significant from monounsaturated (MUFAs) group, d: significant from polyunsaturated (PUFAs) group, e: significant from medium chain (MCFAs) group. significance: p < 0.05. SCFAs: short chain fatty acids, BMI: body mass index.

### Changes in liver injury markers; ALT and AST in different groups

Table 2 shows that serum alanine transaminase (ALT) and aspartate transaminase (AST) were significantly increased in the SFD group as compared to other groups. The differences between levels of serum liver enzymes in (MUFAs, PUFAs, and SCFAs) groups were insignificant, whereas the level in the MCFAs group was significantly higher than each of them.

**Table 2:** Serum liver transaminases and lipid profile in different groups.

Groups (n = 7)						
Parameters	Control	SFD	MUFAs	PUFAs	MCFAs	SCFAs
ALT (U/L)	38.49 ± 0.32	123.94^a^ ± 1.51	49.21^ab^ ± 1.43	47.42^ab^ ± 1.47	79.81^abcd^ ± 1.9	52.05^abe^ ± 1.61
AST (U/L)	83.91 ± 0.5	269.91^a^ ± 2.21	107.46^ab^ ± 2.37	102.91^ab^ ± 2.28	155.29^abcd^ ± 4.8	106.29^abe^ ± 1.95
TC (mmol/L)	1.75 ± 0.03	2.88^a^ ± 0.02	2.06^ab^ ± 0.03	2.04^ab^ ± 0.04	2.46^abcd^ ± 0.02	2.04^abe^ ± 0.03
TG (mmol/L)	0.91 ± 0.02	1.51^a^ ± 0.01	1.2^ab^ ± 0.02	1.2^ab^ ± 0.02	1.32^abcd^ ± 0.02	1.22^abe^ ± 0.02
HDL-c (mmol/L)	0.84 ± 0.01	0.54^a^ ± 0.01	0.72^ab^ ± 0.02	0.73^ab^ ± 0.02	0.62^abcd^ ± 0.01	0.7^abe^ ± 0.02
LDL-c (mmol/L)	0.61 ± 0.02	1.83^a^ ± 0.01	0.94^ab^ ± 0.02	0.91^ab^ ± 0.01	1.4^abcd^ ± 0.01	0.93^abe^ ± 0.01

Data represent mean ± S.E of observations from seven animals.

a: significant difference from the control group, b: significant difference from long chain-saturated (SFD) group, c: significant difference from monounsaturated (MUFAs) group, d: significant difference from polyunsaturated (PUFAs) group, e: significant difference from medium chain (MCFAs) group. Significance: p < 0.05. ALT: alanine transaminase, AST: aspartate transaminase, TC: total cholesterol, TG: triglycerides, HDL-c: high density lipoprotein cholesterol, LDL-c: low density lipoprotein cholesterol, SCFAs: short chain fatty acids. n: number of rats in each group.

### Changes in serum lipid profile; TC, TG, HDL-c, and LDL-c in different groups

Serum total cholesterol level (TC), triglycerides (TG), and low-density lipoprotein-cholesterol (LDL-c) were significantly increased, while serum high-density lipoprotein-cholesterol (HDL-c) level was decreased in the SFD as compared to other groups. The differences in the serum lipid profile of (MUFAs, PUFAs, and SCFAs) groups were insignificant. Serum TC, TG, and LDL-c levels in the MCFAs group were significantly higher while its serum HDL-c was significantly lower than the levels in (MUFAs, PUFAs, and SCFAs) groups (Table 2).

### Changes in metabolic parameters “fasting serum glucose, insulin, and HOMA-IR” in different groups

Serum glucose, insulin, and HOMA-IR were significantly increased in the SFD group as compared to other groups. The differences in the serum glucose of (MUFAs, PUFAs, and SCFAs) groups were insignificant. The serum glucose level in the MCFAs group was significantly higher than the level in the MUFAs and PUFAs groups, but insignificantly different from its level in the SCFAs group. As regards the differences in the fasting serum insulin and HOMA-IR levels in the MUFAs, PUFAs, MCFAs, and SCFAs groups, they were insignificantly different (Table 3).

**Table 3:** Serum metabolic parameters and adipokines in different groups.

Groups (n = 7)						
Parameters	Control	SFD	MUFAs	PUFAs	MCFAs	SCFAs
Fasting glucose (mg/dl)	90.04 ± 0.8	141.61^a^ ± 1.61	116.14^ab^ ± 0.8	116.66^ab^ ± 1.4	120.6^abcd^ ± 1	117.9^ab^ ± 0.55
Fasting insulin (µU/ml)	9.86 ± 0.34	22^a^ ± 1.59	16^ab^ ± 0.82	16.01^ab^ ± 1.18	17.21^ab^ ± 0.89	16.43^ab^ ± 0.35
HOMA-IR	2.19 ± 0.09	7.72^a^ ± 0.64	4.61^ab^ ± 0.27	4.61^ab^ ± 0.38	5.14^ab^ ± 0.31	4.78^ab^ ± 0.12
Leptin (pg/ml)	242 ± 1.88	378.14^a^ ± 3.01	278.71^ab^ ± 2.87	276.14^ab^ ± 3.63	312.29^abcd^ ± 6.01	282.14^abe^ ± 2.98
Adiponectin (ng/ml)	8.87 ± 0.1	4.49^a^ ± 0.07	7.3^ab^ ± 0.24	7.63^ab^ ± 0.19	6.13^abcd^ ± 0.04	7.33^abe^ ± 0.15
Adiponectin/leptin ratio (pg/pg)	36.6 ± 0.13	11.86^a^ ± 0.1	26.14^ab^ ± 0.64	27.6^abc^ ± 0.38	19.66^abcd^ ± 0.26	25.96^abde^ ± 0.28

Data represent mean ± S.E. of observations from seven animals. a: significant from the control group. b: significant from long saturated fat (SFD) group, c: significant from monounsaturated (MUFAs) group, d: significant from polyunsaturated (PUFAs) group, e: significant from medium chain (MCFAs) group. significance: p < 0.05. SCFAs: short chain fatty acids, HOMA-IR: Homeostatic Model Assessment for insulin resistance.

### Changes in serum adipokines “leptin and adiponectin”, and adiponectin/leptin ratio in different groups

Table 3 shows that serum leptin was significantly increased, while serum adiponectin and adiponectin/leptin ratio were significantly decreased in the SFD group as compared to other groups. The differences in the serum leptin and adiponectin of the MUFAs, PUFAs, and SCFAs groups were insignificant. Serum leptin level in the MCFAs group was significantly higher while its serum adiponectin level was significantly lower than the levels in the MUFAs, PUFAs, and SCFAs groups. As regards the differences in the serum adiponectin/leptin ratio, it had no significant difference between MUFAs and SCFAs groups. The ratio in the PUFAs group was significantly higher than levels in the MUFAs, MCFAs, and SCFAs groups. The ratio in the MCFAs group was significantly lower than the ratio in the MUFAs, PUFAs, and SCFAs groups.

### Changes in pro-inflammatory markers; MDA and IL-6 and anti-inflammatory markers; TA and IL-10 in different groups

Data presented in Table 4 show that serum malondialdehyde (MDA) and interleukin-6 (IL-6) levels were significantly increased, while serum total anti-oxidants (TA) and interleukin-10 (IL-10) levels were significantly decreased in the SFD group as compared to other groups. The differences in the serum MDA, IL-6, TA, and IL-10 between (MUFAs, and SCFAs) groups were insignificant. Serum MDA and IL-6 levels in the PUFAs group were significantly lower, while its serum TA and IL-10 levels were significantly higher than levels in the MUFAs, MCFAs, and SCFAs groups. The MCFAs group had significantly lower levels of TA and IL-10, while its serum MDA and IL-6 levels were significantly higher than the levels in the MUFAs, PUFAs, and SCFAs groups. The PUFAs group showed the greatest antioxidant and anti-inflammatory effects.

**Table 4:** Serum pro-inflammatory and anti-inflammatory markers in different groups.

Groups (n = 7)						
Parameters	Control	SFD	MUFAs	PUFAs	MCFAs	SCFAs
MDA (nmol/ml)	4.86 ± 0.16	12.12^a^ ± 0.12	6.74^ab^ ± 0.1	5.71^abc^ ± 0.13	8.81^abcd^ ± 0.12	6.42^abde^ ± 0.12
TA (µmol/L)	1.76 ± 0.03	0.78^a^ ± 0.02	1.21^ab^ ± 0.01	1.62^abc^ ± 0.03	0.99^abcd^ ± 0.02	1.19^abde^ ± 0.01
IL-6 (pg/ml)	34.14 ± 0.8	136.14^a^ ± 2.28	71.86^ab^ ± 2.74	58.43^abc^ ± 1.73	82.14^abcd^ ± 2.33	66^abde^ ± 2.45
IL-10 (pg/ml)	134.93 ± 0.68	72.43^a^ ± 2.66	92.21^ab^ ± 0.94	108.71^abc^ ± 2.53	83.5^abcd^ ± 0.49	92.5^abde^ ± 0.92

Data represent mean ± S.E of observations from seven animals.

a: significant difference from the control group, b: significant difference from long chain-saturated (SFD) group, c: significant difference from monounsaturated (MUFAs) group, d: significant difference from polyunsaturated (PUFAs) group, e: significant difference from medium chain (MCFAs) group. Significance: p < 0.05. MDA: malondialdehyde, TA: Total anti-oxidants, IL-6: interleukin-6, IL-10: interleukin-10, SCFAs: short chain fatty acids. n: number of rats in each group.

### Histopathological and immuno-histochemical results

#### Histopathological results of the liver (H&E and Masson’s trichrome stains) (Figs. 1, 2; Table 5)

**Figure 1:**
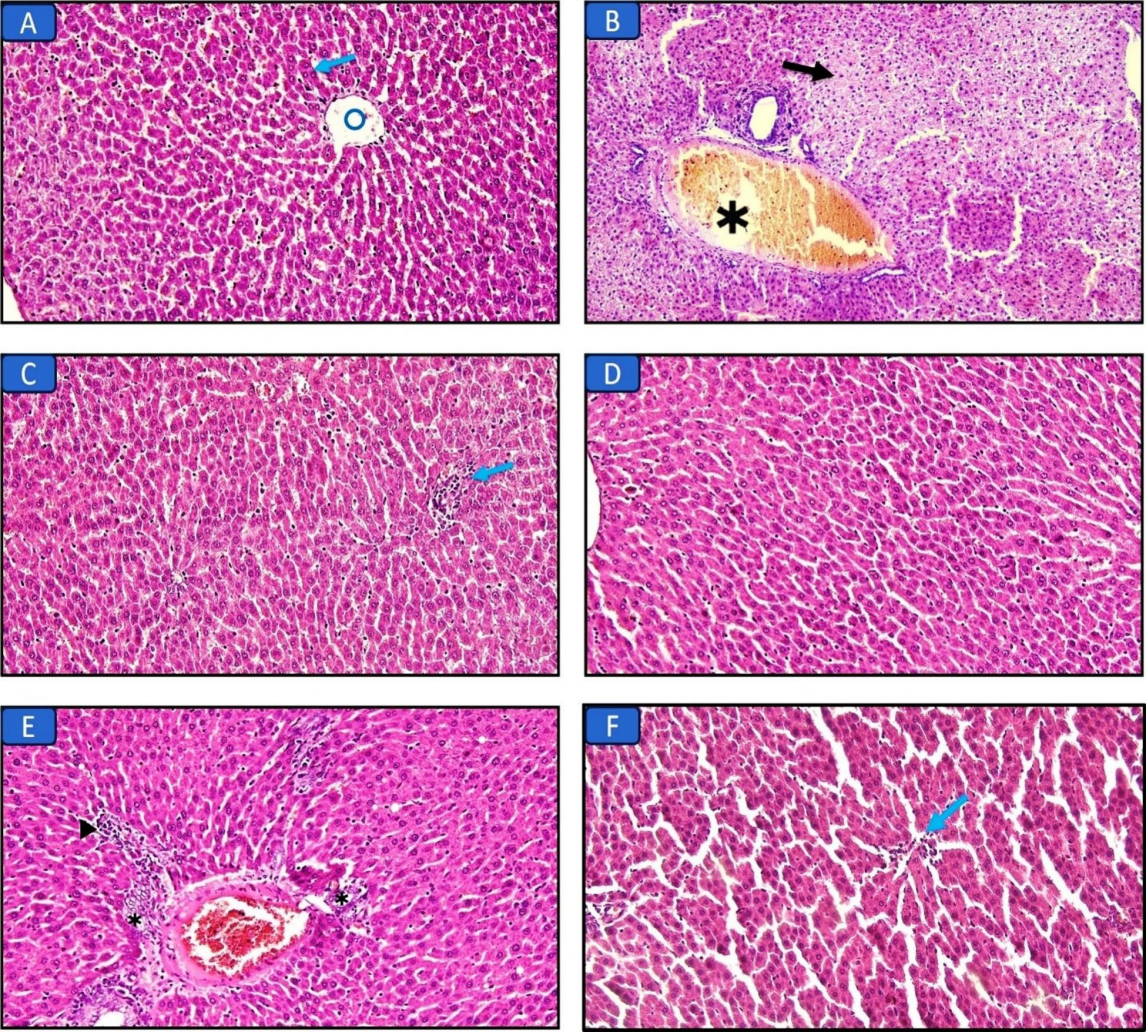
Hematoxylin and eosin-stained liver sections in the different groups: (A) Control group, normal liver structure with central vein (circle) and hepatocytes (arrow) (×200). (B) Long chain saturated fat diet group, the liver shows diffuse injury with necrosis (arrow) and congestion (asterisk) (×200). (C) Long chain monounsaturated fat diet group, the liver shows focal mild lymphocytic infiltrate (arrow) (×200). (D) Long chain polyunsaturated fat diet group, the liver structure was normal regarding hepatocytes and bile canaliculi (×200). (E) Medium chain fat diet group, the liver shows bile duct proliferation (asterisks) and focal mild lymphocytic infiltrate (arrowhead) (×200). F) Short chain fat diet group, the liver shows focal mild lymphocytic infiltrate (arrow) (×200).

**Figure 2:**
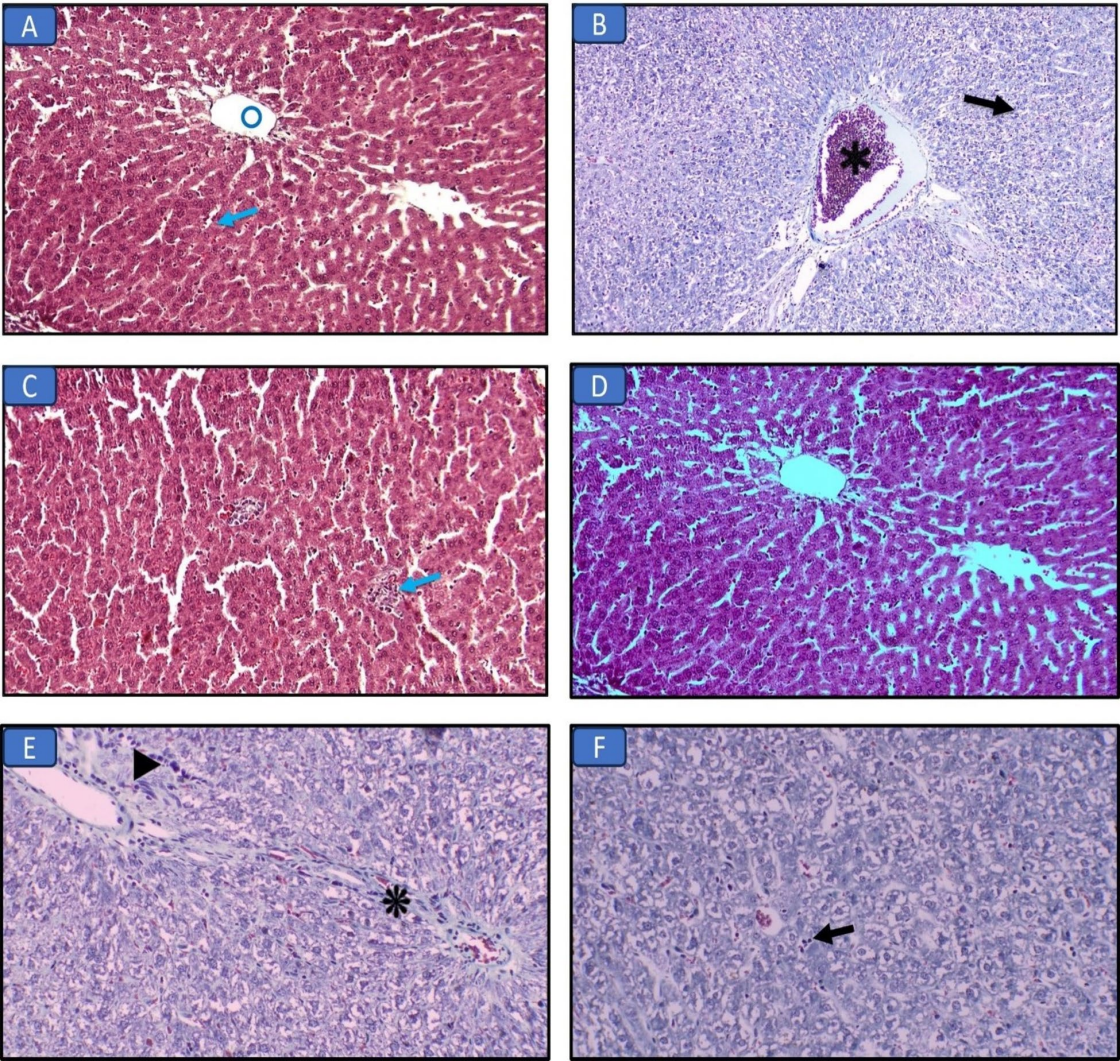
Masson’s trichrome-stained liver sections in the different groups: (A) Control group, normal liver structure with central vein (circle) and hepatocytes (arrow) (×200). (B) Long chain saturated fat diet group, the liver shows diffuse injury with necrosis (arrow) and congestion (asterisk) (×200). (C) Long chain monounsaturated fat diet group, the liver shows focal mild lymphocytic infiltrate (arrow) (×200). (D) Long chain polyunsaturated fat diet group, the liver structure was normal regarding hepatocytes and bile canaliculi (×200). (E) Medium chain fat diet group, the liver shows fibrous tissue band (asterisk) and focal mild lymphocytic infiltrate (arrowhead) (×200). (F) Short chain fat diet group, the liver shows focal mild lymphocytic infiltrate (arrow) (×200).

**Table 5:** Histopathological evaluation of the liver tissue of all groups.

Parameters	Control	SFD	MUFAs	PUFAs	MCFAs	SCFAs
Inflammation	0	2	1	0	1	1
Necrosis	0	3	0	0	1	0
Ballooning	0	3	1	1	2	1
Bile duct proliferation	0	1	0	0	2	0
Fibrosis	0	2	0	0	1	0
Hemorrhage	0	2	0	0	0	0
Total scoring	0	13^a^ ± 0.87	2^ab^ ± 0.31	1^ab^ ± 0	7^abcd^ ± 0.49	2^abe^ ± 0.31

Data represent mean ± S.E. of observations from seven animals. a: significant from the control group. b: significant from long saturated fat (SFD) group, c: significant from monounsaturated (MUFAs) group, d: significant from polyunsaturated (PUFAs) group, e: significant from medium chain (MCFAs) group. significance: p < 0.05. SCFAs: short chain fatty acids.

Liver sections of the control group showed normal liver structure with central vein and hepatocytes. Examination of liver sections in the SFD group showed diffuse injury with necrosis and congestion. While, other groups that received MUFAs, MCFAs, or SCFAs diets showed an improvement regarding the hepatic structure with focal mild lymphocytic infiltration. PUFAs diet group showed normal hepatic architecture and bile canaliculi.

#### Immunohistochemical staining of Nrf2 in the liver tissue of all groups (Fig. 3; Table *7)

**Figure 3:**
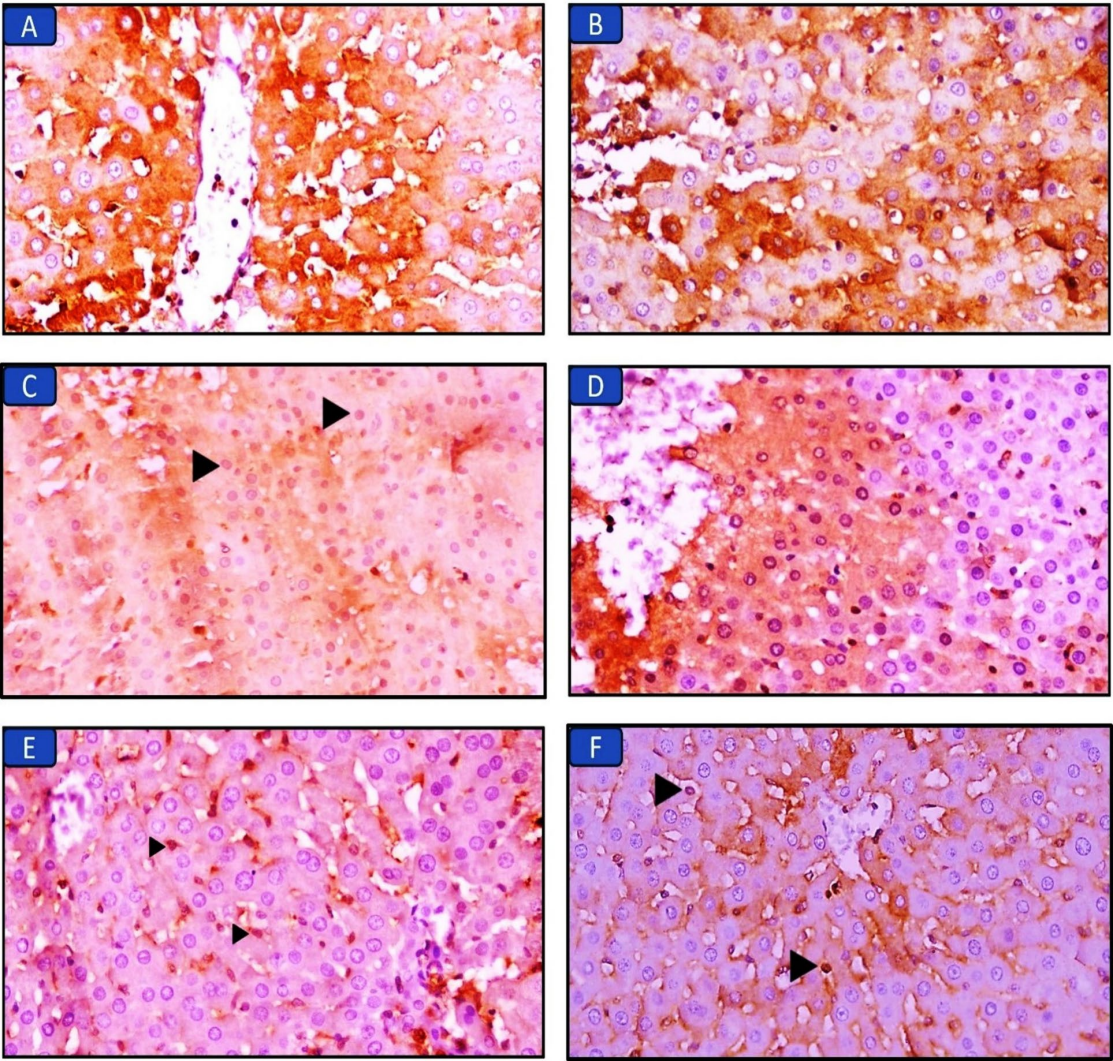
Immuno-expression of Nrf2 in liver sections in the different groups: (A) Control group, marked cytoplasmic expression in hepatocytes (×400). (B) Long chain saturated fat diet group, the liver shows moderate cytoplasmic expression in hepatocytes (×400). (C) Long chain monounsaturated fat diet group, the liver shows mild cytoplasmic and moderate nuclear expression in hepatocytes (arrowheads) (×400). (D) Long chain polyunsaturated fat diet group, the liver shows mild cytoplasmic and marked nuclear expression in hepatocytes (×400). (E) Medium chain fat diet group, the liver shows mild nuclear (arrowheads) and cytoplasmic expression in hepatocytes (×400). (F) Short chain fat diet group, the liver shows moderate nuclear expression (arrowheads) and mild cytoplasmic expression in hepatocytes (×400).

Liver sections of the control group showed the normal cytoplasmic expression of Nrf2. The groups that received MUFAs, MCFAs, or SCFAs diets showed mild cytoplasmic expression of Nrf2 with mild to moderate nuclear expression (scoring: 2–3). Liver sections of the PUFAs diet group showed mild cytoplasmic expression associated with marked nuclear expression (scoring: 4). On the contrary, the SFD group had minimal or no nuclear expression of Nrf2 (scoring: 0–1).

#### Histopathological results of the aorta (H&E and Masson’s trichrome stains) (Figs. 4, 5; Table 6)

**Figure 4:**
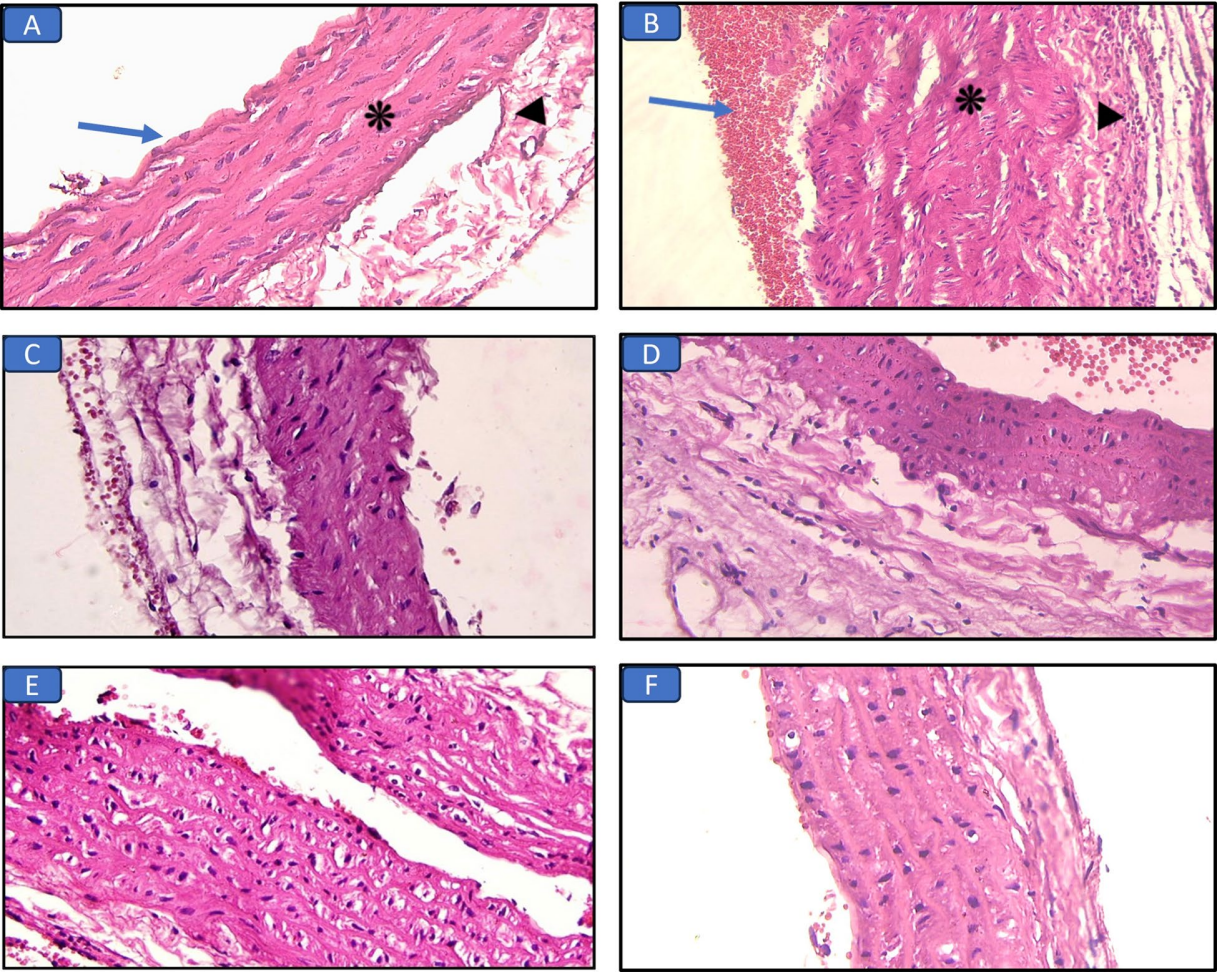
Hematoxylin and eosin-stained aorta sections in the different groups: (A) Control group, the aorta showed continuous layer of endothelial at the tunica intima (arrow) and normal thickness of the tunica media (asterisk) and adventitia (arrowhead) (×400). (B) Long chain saturated fat diet group, the aorta shows marked tunica media thickening (asterisk) with congestion (arrows) and lymphocytic infiltration in the adipose tissue around aorta (arrowhead) (×400). (C) Long chain monounsaturated fat diet group, the aorta shows mild intimal and media thickening (×400). (D) Long chain polyunsaturated fat diet group, normal aorta structure (×400). (E) Medium chain fat diet group, the aorta shows moderate intima and media thickening (×400). (F) Short chain fat diet group, normal aorta structure (×400).

**Figure 5:**
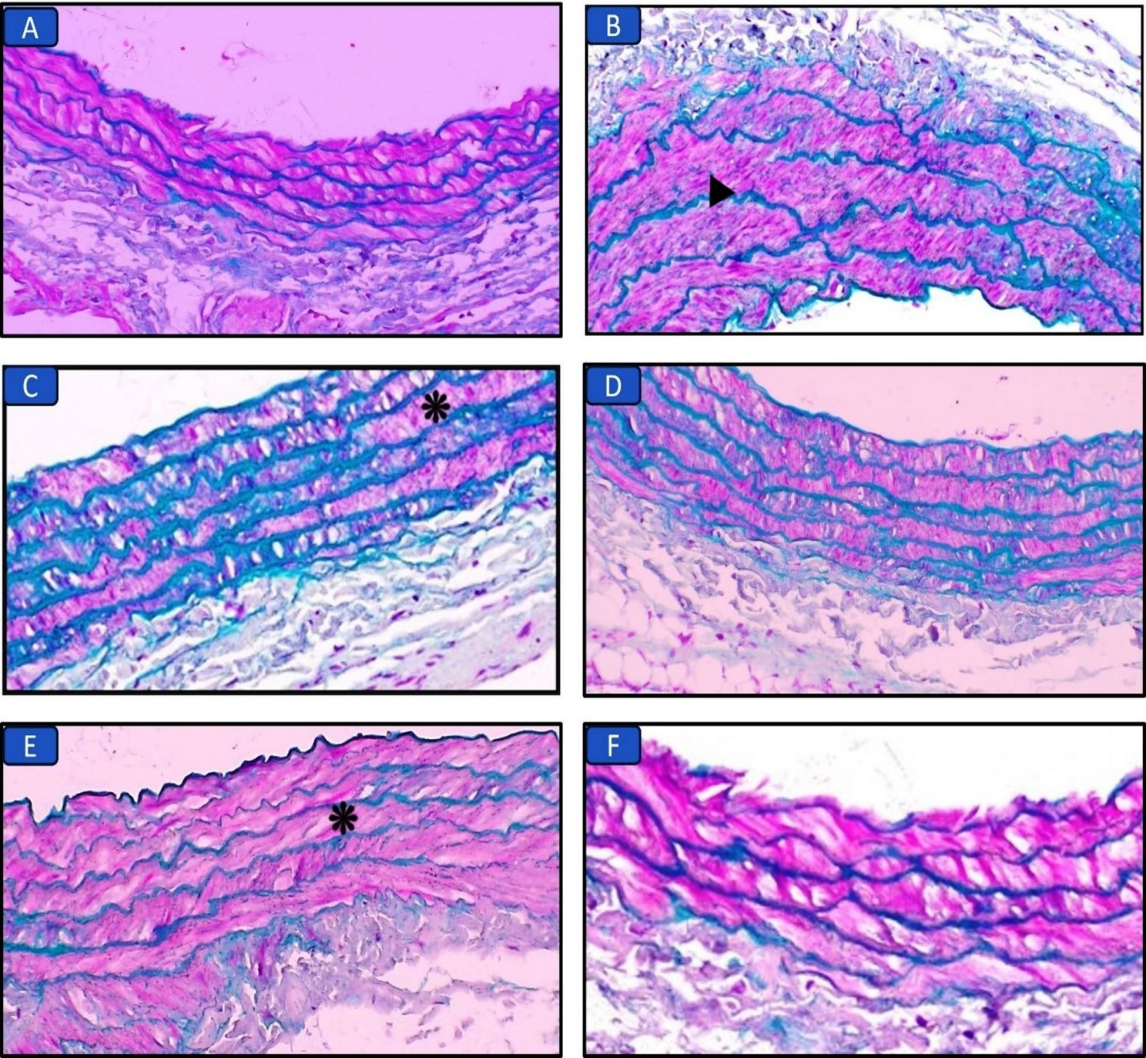
Masson’s trichrome-stained aorta sections in the different groups: (A) Control group, normal aorta structure (×400). (B) Long chain saturated fat diet group, the aorta shows marked media thickening and elastosis (arrowhead) (×400). (C) Long chain monounsaturated fat diet group, the aorta shows mild intima and media thickening (asterisk) (×400). (D) Long chain polyunsaturated fat diet group, normal aorta structure (×400). (E) Medium chain fat diet group, the aorta shows moderate media thickening (asterisk) (×400). (F) Short chain fat diet group, normal aorta structure (×400).

**Table 6:** Histopathological evaluation of the aorta of all groups.

Parameters	Control	SFD	MUFAs	PUFAs	MCFAs	SCFAs
Media thickening	0	3	1	0	2	0
Inflammatory infiltration around aorta	0	2	1	1	0	2
Elastosis	0	1	0	0	1	0
Total scoring	0	6^a^ ± 0.53	2^ab^ ± 0.49	1^ab^ ± 0.22	3^abd^ ± 0.49	2^ab^ ± 0.22

Data represent mean ± S.E. of observations from seven animals. a: significant from the control group. b: significant from long saturated fat (SFD) group, c: significant from monounsaturated (MUFAs) group, d: significant from polyunsaturated (PUFAs) group, e: significant from medium chain (MCFAs) group. significance: p < 0.05. SCFAs: short chain fatty acids.

The control group showed normal aorta structure. Examination of aorta sections in the SFD group showed marked media thickening, congestion, elastosis, and lymphocytic infiltration in the adipose tissue around aorta. While, other groups that received MUFAs, or MCFAs diets showed mild to moderate intima and media thickening. SCFAs and PUFAs diet groups showed normal aortic structure.

#### Immunohistochemical staining of Nrf2 in the aorta of all groups (Fig. 6; Table 7)

**Figure 6:**
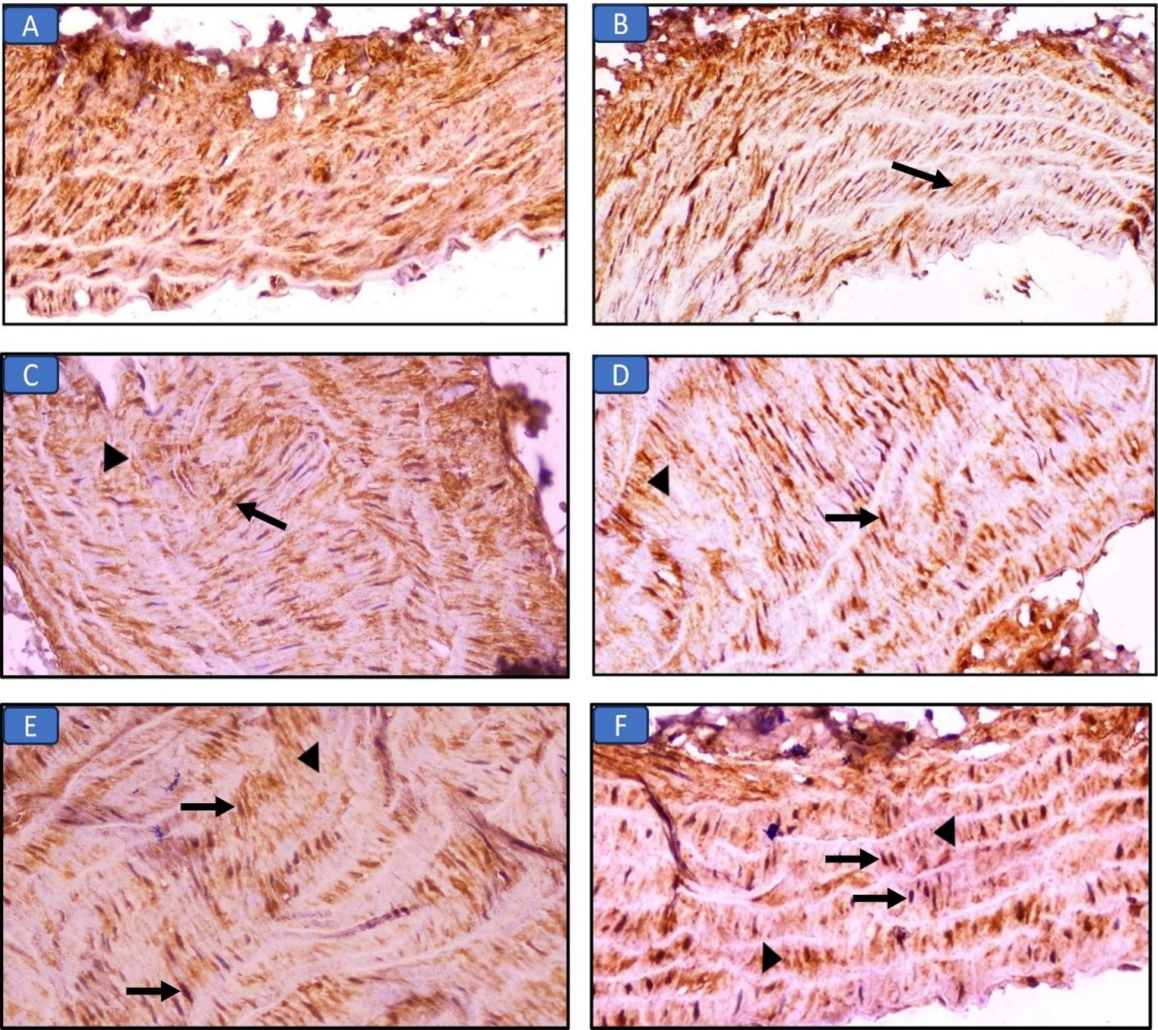
Immuno-expression of Nrf2 in aorta sections in the different groups: (A) Control group, the aorta shows marked cytoplasmic expression in muscle layer (×400). (B) Long chain saturated fat diet group, the aorta shows moderate cytoplasmic expression in tunica media (arrow) (×400). (C) Long chain monounsaturated fat diet group, the aorta shows moderate nuclear (arrow) and mild cytoplasmic (arrowhead) expression in tunica media (×400). (D) Long chain polyunsaturated fat diet group, the aorta shows marked nuclear (arrow) and mild cytoplasmic (arrowhead) expression in tunica media (×400). (E) Medium chain fat diet group, the aorta shows mild nuclear (arrows) and cytoplasmic (arrowhead) expression in muscle layer (×400). (F) Short chain fat diet group, the aorta shows moderate nuclear (arrows) and mild cytoplasmic (arrowheads) expression in tunica media (×400).

**Table 7:** Immunohistochemical evaluation (cytoplasmic and nuclear expression) of Nrf2 in liver and aorta tissues of all groups.

Tissue	Expression	Control	SFD	MUFAs	PUFAs	MCFAs	SCFAs
Liver	Nuclear	0	0	2^ab^	4^abc^	1^abcd^	2^abde^
	Cytoplasmic	3	2^a^	1^ab^	1^ab^	1^ab^	1^ab^
Aorta	Nuclear	0	0	2^ab^	4^abc^	1^abcd^	2^abde^
	Cytoplasmic	3	2^a^	1^ab^	1^ab^	1^ab^	1^ab^

Data represent mean ± S.E of observations from seven animals. MUFAs: monounsaturated group, PUFAs: polyunsaturated group, MCFAs: Medium chain group, SCFAs: short chain group. Significance when P < 0.05. a: significant from control, b: significant from long chain saturated (SFD) group, c: significant from MUFAs group, d: significant from PUFAs group, e: significant from MCFAs group.

Aorta sections of the control group showed the normal cytoplasmic expression of Nrf2. The groups that received MUFAs, MCFAs, or SCFAs diets showed mild cytoplasmic expression of Nrf2 with mild to moderate nuclear expression (scoring: 2–3). PUFAs diet group showed mild cytoplasmic expression with marked nuclear expression (scoring: 4) in the sections of the aorta, while, the SFD group had minimal or no nuclear expression of Nrf2 (scoring: 0–1).

## Discussion

In this study, we aimed to evaluate the impact of the consumption of different types of high-caloric fat diets on the metabolic status and the integrity of the liver and aorta in rats and to explore whether all types of high-fat diets are dangerous, and which ones can potentially guard against the hazards of high caloric diet.

It was documented that consuming a diet rich in long-chain saturated fat led to an evident increase in body weight as a result of lipogenesis enhancement and impairment of energy expenditure^16^*.* In the present study, this was indicated by the significant increase in final body mass index (BMI) and Lee index in the long-chain saturated fat diet (SFD) group, compared to the control group and other high-fat diet (HFD) groups. Moreover, this group showed a significant increase in the amount of visceral fat, which had an imperative impact on the metabolic status^17^*.* This was not the case in the other HFD groups that received diets rich in monounsaturated fatty acids (MUFAs), polyunsaturated fatty acids (PUFAs), medium chain fatty acids (MCFAs), or short chain fatty acids (SCFAs) as they showed significantly lower levels regarding the final BMI, Lee index and visceral fat. This may be due to their satiating effect, and enhancing fatty acid oxidation^18^.

Increased visceral adiposity was reflected on the metabolic status, as the SFD group showed elevated serum levels of fasting glucose, fasting insulin, and HOMA-IR levels when compared to other groups indicating a state of insulin resistance. The visceral adiposity, oxidative stress, adipose tissue dysfunction, chronic inflammation, and dyslipidemia could explain the insulin resistance seen in this group. Hyperinsulinemia occurs firstly to compensate for the reduced sensitivity of cells to insulin. This compensatory mechanism aims to maintain the blood glucose level, but it also contributes to various metabolic abnormalities^19^*.* On the contrary, insulin resistance was significantly lower in MUFAs, PUFAs, MCFAs, and SCFAs groups. This result is in agreement with previous studies^20–23^*.* This could be explained by their protective mechanisms including anti-inflammatory, anti-oxidant, and adiposity-lowering effects which were significantly more obvious in the group that received PUFAs.

There is a strong association between insulin resistance induced by obesity and dyslipidemia, as shown in the SFD group. There was a significant increase in the serum levels of TC, TG, and LDL-c, and a significant decrease in the serum level of HDL-c as compared to the control group and other groups. This may be due to down-regulation of LDL receptors on cell surfaces. This result was supported by the previous studies done by Mamun et al. and Yasmin et al.^24,25^*.* The dyslipidemic profile was significantly lower in groups that received MUFAs, PUFAs, MCFAs, and SCFAs. They up-regulate LDL receptors, protect LDL particles from oxidative stress, and contribute to the formation and maturation of HDL particles in the bloodstream which has a defensive influence on the aortic structure as seen in the histopathological images^26^*.*

Excessive fat accumulation, especially in the visceral compartment, endorses the production and accumulation of reactive oxygen species (ROS). This accumulation in turn leads to redox imbalances and an oxidative status^27^*.* This was the case in the SFD group as it showed significantly higher levels of oxidative stress at the biochemical and histopathological picture as compared to the control group and other HFD groups. This was indicated by the significantly higher serum level of malondialdehyde (MDA) and lower serum level of total antioxidants (TA), along with a significant decrease in the nuclear immune expression of Nuclear Factor Erythroid-2-Related Factor-2 (Nrf2) in both hepatic and aortic tissues. Nrf2 is a known transcription factor that translocates into the nucleus for expression of anti-oxidative genes. These results are compatible with previous experiments conducted by Ma, Tamer et al., and Tan et al.^28–30^*.* The previous results may be due to consumption of the available anti-oxidants during the fight against oxidative damage, and hepatic oxidative stress subsequently leads to damage of hepatic cell membranes with consequential release of the liver enzymes in the circulation as that was mentioned by Bhandarkar et al. and Nanizawa et al.^31,32^. The occurrence of hepatocellular damage in this group was indicated by elevated liver enzymes, along with disturbed hepatic architecture as seen in the histopathological images that showed diffuse hepatic injury and hemorrhage.

Groups that received diets rich in MUFAs, PUFAs, MCFAs, or SCFAs showed a significant improvement regarding oxidative damage. This was indicated by the significant elevation in the serum level of TA, and the significant decrease in the serum level of liver enzymes and MDA in these groups. Notably, the group that received PUFAs had the highest anti-oxidant effect, as PUFAs have a direct ROS scavenger effect. Moreover, these results were in agreement with the significant improvement in the hepatic and aortic architecture in the histopathological images, and the significant increase in the nuclear immune expression of Nrf2, indicating the anti-oxidative impact. These results come in line with previous studies^33–36^. The effect of chain length and saturation on oxidative stress was reported by Xu et al.^37^ which supported the highest anti-oxidative effect of PUFAs compared to the lowest effect of MCFAs among the previous groups.

The oxidative stress shown in the SFD group was associated with an increased inflammatory status. This was indicated by increased serum levels of the proinflammatory IL-6 and decreased serum level of the anti-inflammatory IL-10 associated with the noticed increased inflammatory cell infiltration in both the hepatic and aortic tissues. Moreover, chronic inflammation may lead to adipose tissue dysfunction. This was characterized by an increased serum level of leptin and a decreased serum level of adiponectin and adiponectin/leptin ratio. Adiponectin is consumed in combating pro-inflammatory mediators as seen here. These results are in agreement with experiments conducted by Sergi and Williams and Mendoza-Herrera et al.^38,39^*.*

The inflammatory status was less obvious in MUFAs, PUFAs, MCFAs, and SCFAs groups, as there was an increase in the anti-inflammatory IL-10, and the anti-inflammatory adipokine, adiponectin with a decrease in the pro-inflammatory markers; IL-6 and leptin, with a noticeable decreased lymphocytic infiltration in the aortic and hepatic tissues. These results are consistent with experiments conducted by Montserrat-de la Paz et al., Yang et al., and Żebrowska et al.^40–42^*.* The group that received PUFAs showed the highest anti-inflammatory effect, the result was indicated by the highest level of the adiponectin/leptin ratio. This ratio is a more accurate marker for adipose tissue dysfunction than leptin or adiponectin levels alone^43^*.*

Chronic inflammation always leads to fibrosis as a late destination. As seen here in the histopathological images of liver sections stained with Masson’s Trichrome, the SFD group had significant hepatic fibrosis as compared to the control and other HFD groups which showed less obvious fibrotic changes. This is due to the activation of hepatic stellate cells that produce excessive amounts of extracellular matrix leading to hepatic fibrosis which is the final and common pathological outcome. This result was reported in a previous study conducted by Jia et al.^44^. Moreover, the buildup of inflammatory cells in the endothelium can disrupt its normal function, including altered vasomotor function. This is a critical factor in the development and progression of atherosclerosis of arteries including the aorta^8^. This was indicated by a marked intimal sclerosis and elastosis seen in Masson-stained aortic sections of the SFD group compared to other groups.

Taken all together, it is obviously that all the previous steps including insulin resistance, adipose tissue dysfunction, hepatic steatosis, dyslipidemia, oxidative stress, and the chronic inflammatory status are intermingled in a vicious circle that ultimately disrupt and affect the systemic metabolic status^45^.

## Conclusions

According to our reported data, we concluded that not all types of fat are dangerous. Chain length and saturation degree of fatty acids are crucial factors that impact health. Diets rich in long-chain saturated fat have several hazards to the metabolic status, the liver and aortic health. Its restriction is recommended to protect against oxidative inflammatory status that triggers several metabolic problems including steatohepatitis, hepatic fibrosis, adipose tissue dysfunction, insulin resistance, atherosclerosis, and maybe metabolic syndrome development. On the contrary, diets rich in MUFAs, PUFAs, MCFAs, or SCFAs have beneficial impacts on health. This could be attributed to their anti-inflammatory and anti-oxidative effects which were reflected in the improvement of insulin sensitivity that has several metabolic impacts.

## Materials and methods

### Animals and ethical approval

For the whole duration of the current study, 42 adult male Wistar albino rats weighing between 130 and 150 g were utilized. Rats were housed at room temperature (18–26 °C) in regular day/night cycles, which equated to roughly 12 h of day and 12 h of night. They left to acclimatize to the environment for 1 week before their inclusion in the experiment. They were given tap water and a commercial rat chow diet as their standard diet. Mean initial and final body mass index (BMI) were recorded just before the start and at the end of the experiment. The principles of laboratory animal care were followed according to the NIH Guidelines for the Care and Use of Laboratory Animals, and the experimental procedures used in this study were approved by the Animal Care and Use Committee of the Faculty of Medicine, Minia University, Egypt. Approval No. (103:10/2021) that follows the ARRIVE guidelines.

### Experimental groups and diet regimens

The rats were randomly divided into six groups with seven rats each, and given various feeding plans to follow for 9 weeks:

1. *Control group:* This group's rats were fed a standard commercial rat chow diet, which consisted of 21% protein, 3% fat, 48% carbs, and 28% other ingredients like fiber, vitamins, and minerals (Standard diet, SD). According to the manufacturer's information, it provides a diet of roughly 3030 kcal/kg. It was obtained from El-Qahera Company (Minia, Egypt).
2. *Long chain-saturated fat diet group (SFD):* The rats of this group received a high long chain-saturated fat diet (40% fat mainly butter, 30% carbohydrate, 20% protein, and 10% fibers and vitamins). It was made manually, using 38% butter, 6% milk, 6% casein, 2% vitamins, and 48% SD. It provides about 5600 kcal/kg diet^46^*.*
3. *Long chain-monounsaturated fat diet group (MUFAs):* The rats of this group received a high-fat diet rich in olive oil (40% fat mainly olive oil, 30% carbohydrate, 20% protein, and 10% fibers and vitamins). Manually, 48% SD was combined with 30% olive oil, 8% butter, 6% milk, 6% casein and 2% vitamins to make the preparation. It provides about 5600 kcal/kg diet^47^.
4. *Long chain-polyunsaturated fat diet group (PUFAs):* The rats of this group received a high-fat diet enriched with omega-3 (ω − 3) fatty acids. It was made manually by combination of 56% SD with 30% fat “butter”, 6% milk, 6% casein, and 2% vitamins. The ω − 3 fatty acid was given by an oral gavage in a daily dose of 500 mg/kg^48^*.* It provides about 4940 kcal/kg diet.
5. *Medium chain fat diet group (MCFAs):* The rats of this group received a high-fat diet rich in palm oil (40% fat mainly palm oil, 30% carbohydrate, 20% protein, and 10% fibers and vitamins). Manually, 48% SD was mixed with 30% palm oil, 8% butter, 6% milk, 6% casein and 2% vitamins. It provides about 5600 kcal/kg diet^47^.
6. *Short-chain fat diet group (SCFAs):* The rats of this group received a diet enriched with SCFAs. It was prepared manually by combination of 56% SD with 25% fat “butter”, 6% milk, 6% casein, and 2% vitamins. The SCFAs were given by an oral daily dose of 5% wt/wt diet^49^*.* The mixture of SCFAs includes sodium acetate, sodium propionate, and sodium butyrate by the ratio of 12: 5: 3 respectively, it was dissolved in distilled water^50^*.* It provides about 4880 kcal/kg diet.
7. Sodium acetate AR (Anhydrous) was purchased in the form of white coarse powder from Alpha Chemika India. CAS No. 127-09-3
8. Sodium propionate AR was purchased in the form of white powder from Alpha Chemika India. CAS No. 137-40-6
9. Sodium butyrate was purchased in the form of white crystalline hygroscopic powder from Alpha Chemika India. CAS No. 156-54-7

### Calculation of body mass index (BMI), and Lee index

Each rat's body weight was measured at the beginning and end of the experiment. Using a measuring tape, the length of the body was measured from the anus to the base of the central lower incisor at its ventral surface. The body weight and length were estimated to evaluate obesity in rats by the following parameters^51^:

$$ {\text{BMI }} = {\text{ Body weight }}\left( {\text{g}} \right)/{\text{Length}}^{{2}} \left( {{\text{cm}}^{{2}} } \right) $$

$$ {\text{Lee index }} = {\text{ Cube root of body weight }}\left( {\text{g}} \right)/{\text{length }}\left( {{\text{cm}}} \right) $$

### Specimen collection

The rats were decapitated after being anesthetized with urethane at the end of the experiment and following an overnight fast. Jugular vein blood samples were taken, collected into sterile autoclaved tubes, allowed to clot at room temperature, and then centrifuged in a cooling centrifuge for 15 min at 3000 rpm. After that, the serum supernatant was transferred into Eppendorf tubes with labels attached, and it was kept at − 20 °C until it was time to assess various parameters. After that, the abdomen of each rat was opened to collect visceral fat including retroperitoneal fat to be weighed. The liver and aorta were carefully excised, flushed with ice-cold saline, cleaned from blood, and stored in 10% formalin to be used for histopathological and immuno-histochemical examinations. The all obtained liver and thoracic aorta tissues of each group were included in the histopathological examination. The slices were cross sectional.

### Chemical assays

The collected sera were used for estimation of the levels of alanine transaminase (ALT), aspartate transaminase (AST), total cholesterol (TC), triglycerides (TG), HDL cholesterol (HDL-c), total anti-oxidants (TA), glucose, and malondialdehyde (MDA) (Bio-diagnostic Co., EGYPT) by using direct colorimetric method. Serum LDL cholesterol (LDL-c) level was calculated as follows^52^:

$$ {\text{LDL cholesterol conc}}. \, \left( {{\text{mmol}}/{\text{L}}} \right)\, = \,{\text{Total cholesterol}}{-}\left( {{\text{Triglycerides}}/{3}} \right){-}{\text{HDL cholesterol}} $$

Determination of serum levels of interleukin-6 (IL-6), interleukin-10 (IL-10), insulin, leptin, and adiponectin was done by enzyme-linked immunosorbent assay (ELISA) by following the manufacturer protocol (ThermoFisher Scientific Co., USA).

### Calculating homeostasis model assessment of insulin resistance (HOMA-IR) HOMA-IR was determined using the following equation^53^

$$ {\text{HOMA-IR}} = \, \left\{ {{\text{Fasting serum glucose }}\left( {\text{mg/dl}} \right) \, \times {\text{ Fasting serum insulin }}\left( {\upmu {\text{U}}/{\text{ml}}} \right)} \right\}/{4}0{5} $$

### Histopathological procedures

#### Histopathological technique

Following standard histological protocol, the liver and aorta of each animal were removed and preserved in 10% neutral buffered formalin. To identify histological details, tissue sections were prepared, embedded in paraffin wax, and then cut using a microtome to a thickness of 3 μm. They were then stained with hematoxylin and eosin (H&E) and Masson's trichrome stain^54^*.* An Olympus BX50 microscope was used in the current study to evaluate the histopathological alterations at magnifications of 100×, 200× and 400×. The light microscope with a camera connection was used to take pictures.

#### Histopathological scoring

The following parameters were used to evaluate damage to the liver including hepatocyte necrosis, ballooning or degeneration, necrosis, hemorrhage, bile duct proliferation, fibrosis, and the presence of inflammatory cellular infiltration. The following scoring system was used to evaluate tissue injury: zero indicates normal, one indicates minimal damage (less than 10% of hepatocytes in the centrilobular area), two indicates moderate damage (10–50% of hepatocytes in the centrilobular area), and three indicates serious injury (more than 50% of hepatocytes in the centrilobular area)^55^.

The histopathological changes of the aorta were assessed for the following changes, tunica media thickening, elastosis, and inflammatory cell infiltration, and graded as 0, none; 1, mild; 2, moderate; and 3, severe^56,57^.

### Immunohistochemistry:

#### Immunohistochemical technique

Five-micrometer-thick sections were prepared from liver and aorta specimens of different animal groups and immunohistochemistry (IHC) was performed for nuclear factor-erythroid 2 related factor 2 (Nrf2) according to Li et al.^58^ using selective antibodies (Sigma-Aldrich lab, USA).

#### Immunohistochemical scoring of Nrf2

A total of 10 high-power fields were randomly selected and a score was given for each slide. Nrf2 semiquantitative scoring was done by determining immunoreactivity under a light microscope. The extent of staining in the nuclei and cytoplasm was scored as 0 (0%), 1 (1–25%), 2 (26–50%), 3 (51–75%), and 4 (76–100%) according to the percentage of positively stained cells^59,60^*.*

### Statistical analysis of results

Statistical analysis for numerical data was done by SPSS (IBM Corp., Version 20). The mean (M) and standard error (SE) were determined for parameters in each group. The significance of differences observed in these groups was assessed by the Kruskal–Wallis test and post hoc test by Fisher’s Least Significant Difference (LSD) Test. A value of P ≤ 0.05 was considered statistically significant.

### Ethical approval

The experimental protocol and procedures used in this study were approved by the Institutional Ethical Committee of Faculty of Medicine, Minia University, Egypt. (Approval No. 103:10/2021), which follows the NIH Guidelines for the Care and Use of Laboratory Animals and is in accordance with ARRIVE guidelines.

## Data Availability

Data will be available upon request from the corresponding author: [Nardien Ekram Haliem Saleh; Email: nardien_ekram@mu.edu.eg].
